# The impact of DeepSeek’s perceived interactivity on medical students’ self-directed learning ability

**DOI:** 10.1038/s41598-025-33780-3

**Published:** 2026-01-06

**Authors:** Yubin Ju, Jingwei Li, Xiaopeng Zhang, Meijie Wu, Xinyu Pang, Zhengyu Li, Junyang Wang, Jiaxin Li, Yuanyuan Zhang, Xin Dai

**Affiliations:** 1 School of Public Health, Dalian Medical University, Dalian, Liaoning China; 2 Central Hospital of Dalian University of Technology (Dalian Municipal Central Hospital), Dalian, China; 3 The Second Hospital of Dalian Medical University, Dalian, China

**Keywords:** DeepSeek, Perceived interactivity, Self-directed learning ability, Self-efficacy, Medical students

## Abstract

With the rapid advancement of artificial intelligence technology, DeepSeek, as a new-generation generative AI model, has demonstrated significant advantages in the field of medical education. Its robust interactive capabilities and localized deployment features make it particularly well-suited for medical education scenarios. This study aims to explore the mechanism and underlying pathways through which perceived interactivity influences medical students’ self-directed learning ability. It also examines whether social influence indirectly affects self-directed learning ability via the mediating role of self-efficacy, and investigates whether trust moderates the relationship between social influence and behavioral intention. These findings reveal theoretical and practical implications for medical education contexts. This study employed SPSS 27.0 software for statistical data description, utilized Amos 27.0 software to validate the research model, and integrated Process 3.3.1 software to conduct moderation effect analysis. Building upon this foundation, an innovative research framework was constructed by synthesizing three major theoretical models. A random sampling method was used to collect 691 valid questionnaire responses from medical students. Structural equation modeling (SEM) and moderation effect analysis were then applied to test the research hypotheses. Perceived interactivity indirectly promotes willingness to use through performance expectancy (β = 0.180, *p* < 0.001) and effort expectancy (β = 0.428, *p* < 0.001), while social influence exerts the most significant direct effect on willingness to use (β = 0.925, *p* < 0.001). Furthermore, self-efficacy played a crucial mediating role between intention to use and self-directed learning ability (β = 0.575, *p* < 0.001), forming a psychological bridge from technology acceptance to capability enhancement. This study integrates the Unified Theory of Acceptance and Use of Technology (UTAUT), Social Cognitive Theory (SCT), and the Task-Technology Fit (TTF) model to construct a multidimensional mechanism framework examining how perceived interactivity of DeepSeek influences medical students’ autonomous learning capabilities. This study not only validates the synergistic effects of social cognition and technological ease of use in the digital transformation of medical education but also provides theoretical support and practical pathways for the precise adaptation and optimization of DeepSeek within medical education settings. It offers significant implications for advancing the innovative development of medical education.

## Introduction

With the rapid advancement of artificial intelligence technology, search engines have evolved from traditional keyword-matching retrieval tools into intelligent interactive systems centered on generative AI^1^. ChatGPT pioneered a new era of generative AI and has profoundly impacted multiple industries including healthcare, education, and finance^2^. On January 27, 2025, the new generative AI model DeepSeek-R1 officially entered the market and was rapidly adopted, becoming the top-ranked AI application by downloads and demonstrating strong market competitiveness^3^. DeepSeek-R1’s outstanding performance on the CNMLE holds significant implications for the advancement of medical education in China. As an open-source, locally deployable large language model, DeepSeek-R1 enables end-to-end operation in high-privacy scenarios without relying on cloud computing resources. This feature makes it particularly suitable for research and clinical environments with stringent data sovereignty and compliance requirements, such as medical schools and hospital departments. Medical students can leverage DeepSeek-R1 to customize personalized learning paths and simulate real clinical scenarios, thereby enhancing their clinical reasoning abilities^4,5^. This further underscores DeepSeek’s pivotal role in fostering autonomous learning capabilities. As a representative technology in this field, DeepSeek is reshaping the paradigms of information acquisition and knowledge construction.

Against the backdrop of deepening digital transformation, knowledge societies exhibit unprecedented dynamic evolution^6^. The rapid advancement and widespread application of large language models are fundamentally reshaping paradigms for knowledge production, dissemination, and acquisition^7^. This transformation significantly shortens knowledge renewal cycles while driving exponential growth in cognitive processing demands^8^. Generative AI tools like DeepSeek leverage natural language processing and deep learning technologies to generate personalized learning content^9^, simulate clinical decision-making scenarios, and provide instant feedback, thereby enhancing medical students’ knowledge integration and reasoning abilities^2,3^.

Within this complex knowledge ecosystem, autonomous learning capacity serves as a vital bridge connecting individual cognitive development with professional competency enhancement. It has become a core competency for medical and other health science students to adapt to lifelong learning demands, playing a crucial role in their growth and future development^10^. Medical students’ self-directed learning ability is a core competency in the digital age, requiring them to proactively plan, monitor, and evaluate their learning process. Self-directed learning has become the norm for medical students^11^. Autonomous learning has become the norm for medical students^12^. Relevant research indicates that medical students’ autonomous learning abilities can be enhanced to a certain extent through the use of DeepSeek^13^. The application of artificial intelligence technology empowers students to become active participants in the self-directed learning process^14^, thereby fostering deeper understanding, personalized learning, reflection, and collaboration within educational settings^15^. First, teacher support, as a significant external factor^16^, can effectively alter students’ learning strategies and enhance their motivation. Therefore, it is necessary to promptly update existing teaching philosophies and methods to align with the development of the AI education era^17^. Second, technological acceptance capacity is a key factor in enhancing self-regulated learning abilities within generative AI-driven interactive learning environments, presenting new demands and challenges for students’ technological awareness and digital literacy^18^.

In learning environments supported by generative artificial intelligence, interaction, interactivity^19^, and perceived interactivity are related yet distinct concepts. While interchangeable in specific contexts, their underlying meanings warrant careful distinction^20^. Interactivity is typically defined as “human-to-human communication via telecommunication channels, as well as human–computer interaction technologies designed to simulate interpersonal communication”^21^. Perceived interactivity, however, refers to learners’ subjective sense of control over their engagement in the interaction process and their perception of meaningful and engaging responses from the interactive entity^22^. Compared to traditional search engines, DeepSeek demonstrates greater interactive potential. Research indicates that generative AI can enhance learning motivation by boosting interactivity^23^, Against this backdrop, perceived interactivity emerges as a critical factor influencing medical students’ technology adoption and learning outcomes. By enhancing perceived control and feedback immediacy, it increases willingness to use the technology while boosting learning motivation and self-efficacy^24^. As a core feature of DeepSeek, perceived interactivity not only directly enhances learning immersion and engagement but also indirectly fosters self-directed learning capabilities through self-efficacy^25^.

Despite the promising prospects of generative AI in medical education, mechanistic research on how DeepSeek’s perceived interactivity influences medical students’ autonomous learning abilities remains scarce. This study employs a multidimensional theoretical framework, integrating trust and self-efficacy theories, to empirically examine the pathways through which perceived interactivity affects autonomous learning capabilities. The research not only expands the theoretical framework for digital transformation in medical education but also provides practical insights for optimizing DeepSeek’s application in medical education settings.

## Research model and hypotheses

The complexity of digital transformation in medical education makes it difficult for a single theoretical framework to fully explain the multidimensional relationship between technology adoption and learning effectiveness^26^. This study innovatively integrates three major theoretical models—UTAUT, SCT, and TTF. On this basis, trust is introduced as a moderating variable and self-efficacy as a mediating mechanism, thereby establishing a multilevel and multidimensional research framework to systematically reveal the mechanisms through which DeepSeek’s perceived interactivity influences medical students’ self-directed Learning Ability.

The theoretical rationale for integrating these three frameworks lies in the distinctiveness of medical education and the complexity of AI applications. First, the UTAUT model, as the mainstream framework for technology acceptance research, effectively explains users’ willingness to adopt new technologies but has limited explanatory power regarding professional context adaptability and learning outcomes. Second, the TTF model compensates for UTAUT’s shortcomings in task-technology fit and effectively addresses adaptability issues in medical technology applications^27^. Furthermore, the SCT model introduces psychological constructs such as self-efficacy, providing a psychological mechanism for understanding how technology use translates into skill enhancement^28^. In medical education, self-efficacy plays a significant mediating role between technology use and learning outcomes. This finding provides theoretical support for integrating SCT into this study.

**Fig. 1:**
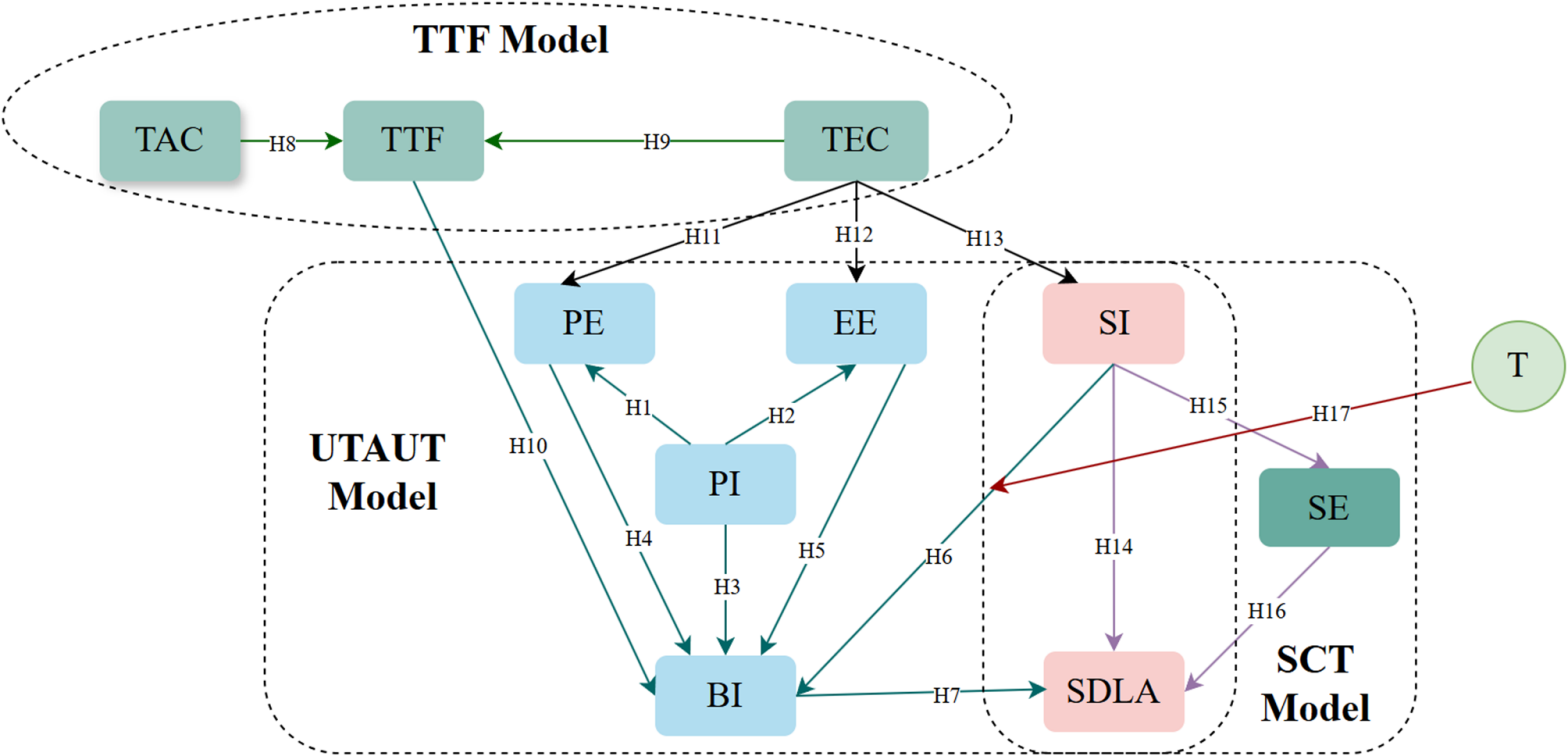
Extended UTAUT-TTF-SCT integrated model.

In summary, the integration of these three theoretical frameworks not only provides a more comprehensive explanatory perspective but also reveals the complex interplay among perceived Interactivity (PI), user perceptions, social environment, task-technology fit (TTF), psychological mechanisms and self-directed learning ability in the digital transformation of medical education. Building upon this foundation, this study proposes the following hypotheses and constructs a corresponding research model, as illustrated in Fig. 1.

### UTAUT model

The UTAUT model integrates key relevant models from the 1990s (particularly TAM, the Technology Acceptance Model), accounting for the relevance of social influence on intention and usage. It comprises four constructs (i.e., effort expectancy, performance expectancy, social factors, and facilitating conditions) and four moderating variables (i.e., age, gender, education, and voluntary usage). These constructs, combined with different moderating variables, directly influence behavioral intention assessment^29^.

The UTAUT model has been widely applied in assessing technology acceptance^30^. In recent years, artificial intelligence technology has undergone continuous iteration and deep penetration into the education sector, giving rise to a series of tools, services, and systems directly usable by end-users. Among these, AI applications primarily focus on key scenarios such as predictive analysis of learning behaviors, personalized recommendation systems, and automated conversational agents (chatbots)^31^.

Performance expectancy(PE) is defined as the degree to which an individual believes that using a specific technology can enhance their work or learning performance. As a key cognitive factor, performance expectancy influences users’ attitudes, which in turn affect their willingness to use the technology and their actual behavior. Scholars across various fields have widely applied performance expectancy to predict users’ attitudes toward and adoption behaviors of technological products^32–35^. In the context of applying DeepSeek within educational settings, effort expectancy(EE) refers to students’ perceptions of its ease of use. Social influence(SI) is recognized as a key factor affecting technology adoption^23,36^. Social influence is typically defined as the support or encouragement for using a specific technology provided by significant others within an individual’s social environment, such as peers, teachers, or other key Figs. ^36^. This influence is crucial in shaping students’ intentions to use technology in educational settings. Existing research indicates that social influence plays a significant role in promoting the adoption of educational technology^37^. Moreover, this model was extended to incorporate perceived trust and perceived risk, revealing these factors to be highly significant. Scholar Mishra conducted research on mobile payments in Portugal, employing social influence and other variables as performance indicators, and found these to be crucial structural elements^38^.

Based on the aforementioned theory, this study proposes the following hypotheses:

- H1: Perceived interactivity positively influences performance expectations.
- H2: Perceived interactivity positively influences effort expectations.
- H3: Perceived interactivity positively influences behavioral intention.
- H4: Performance expectations positively influence behavioral intention.
- H5: Effort expectations positively influence behavioral intention.
- H6: Social expectations positively influence behavioral intention.
- H7: Behavioral intention positively influences self-directed learning ability.

### TTF model

Task-Technology Fit (TTF) was first proposed by Goodhue and Thompson in 1995. It posits that the degree of alignment between the technology users employ and the characteristics of their tasks directly influences their evaluation of technological performance and willingness to use it^39,40^. This theoretical framework measures whether a tool or system’s functionality adequately meets users’ task requirements^41^. Task characteristics(TAC) refer to the attributes and requirements necessary for successfully completing a specific task^42^. These characteristics typically include task complexity, uncertainty, interdependence, and information processing demands^43,44^.

Recent research supports the association between task characteristics and task-technology fit (TTF). Scholars generally agree that learners’ willingness to adopt a technology significantly increases when they perceive it as effectively aiding their daily tasks. This theoretical framework further reveals the practical elements of technology use. The TTF model emphasizes the alignment between technological characteristics(TEC) and task requirements, rather than focusing solely on learners’ subjective expectations of the technology^45^.

Based on the aforementioned theory, this study proposes the following hypotheses:

- H8: Task characteristics positively influence task-technology fit
- H9: Technology characteristics positively influence task-technology fit
- H10: Task-technology fit positively influences willingness to use
- H11: Technology characteristics positively influence performance expectations
- H12: Technology characteristics positively influence effort expectations
- H13: Technology characteristics positively influence social expectations

### SCT model

Social Cognitive Theory (SCT) serves as a core theoretical framework for analyzing human motivation, cognition, and behavior. This theory proposes a causal interaction model where behavior, cognition, and other individual factors interact with environmental influences as mutually determining factors, exerting combined effects through bidirectional mechanisms^46^. Self-efficacy(SE) one of SCT’s central constructs, refers to an individual’s judgment of their ability to organize and execute actions necessary to accomplish a task. Self-efficacy directly influences an individual’s behavioral choices, effort levels, and persistence in the face of obstacles^47^. According to SCT, self-efficacy is regarded as a primary determinant of task performance and has been demonstrated to exert significant psychological and behavioral effects across multiple domains of social-psychological functioning. Empirical research in computer and information technology has shown self-efficacy to be a crucial determinant affecting users’ perceptions of technology and their willingness to adopt it^48^. We believe that the mediating role identified in scholar Priyanka Singh’s research may share a similar underlying logic^49^.

Based on the aforementioned theory, this study proposes the following hypotheses:

- H14: Social expectations positively influence self-directed learning ability.
- H15: Social expectations positively influence systemic self-efficacy.
- H16: Self-efficacy mediates the relationship between social influence and Self-directed learning ability.

### The moderating role of trust

Trust in artificial intelligence is a critical prerequisite for medical students to adopt DeepSeek in their studies, as trust constitutes a core factor in the acceptability of emerging technologies. In generative AI, particularly in the application of DeepSeek for assisted learning, potential users commonly express concerns about risks such as data breaches and system failures. Therefore, robust data protection mechanisms are essential for building user trust^50,51^. In an interdisciplinary context, trust is typically defined as a psychological state wherein individuals are willing to assume vulnerability based on positive beliefs or expectations regarding another party’s behavior or intentions^52^. In the AI domain, understanding the essence of user trust is crucial, as it not only influences perceptions of potential risks and system vulnerabilities but also determines the key factors in trust formation^53^. Furthermore, individuals with higher interpersonal trust levels are more likely to perceive emotional support from teachers, thereby exhibiting stronger academic self-efficacy.

Based on the aforementioned theory, this study proposes the following hypothesis:

- H17: Trust moderates the relationship between behavioral intention and social influence.

## Research methodology

### Program and participants

**Fig. 2:**
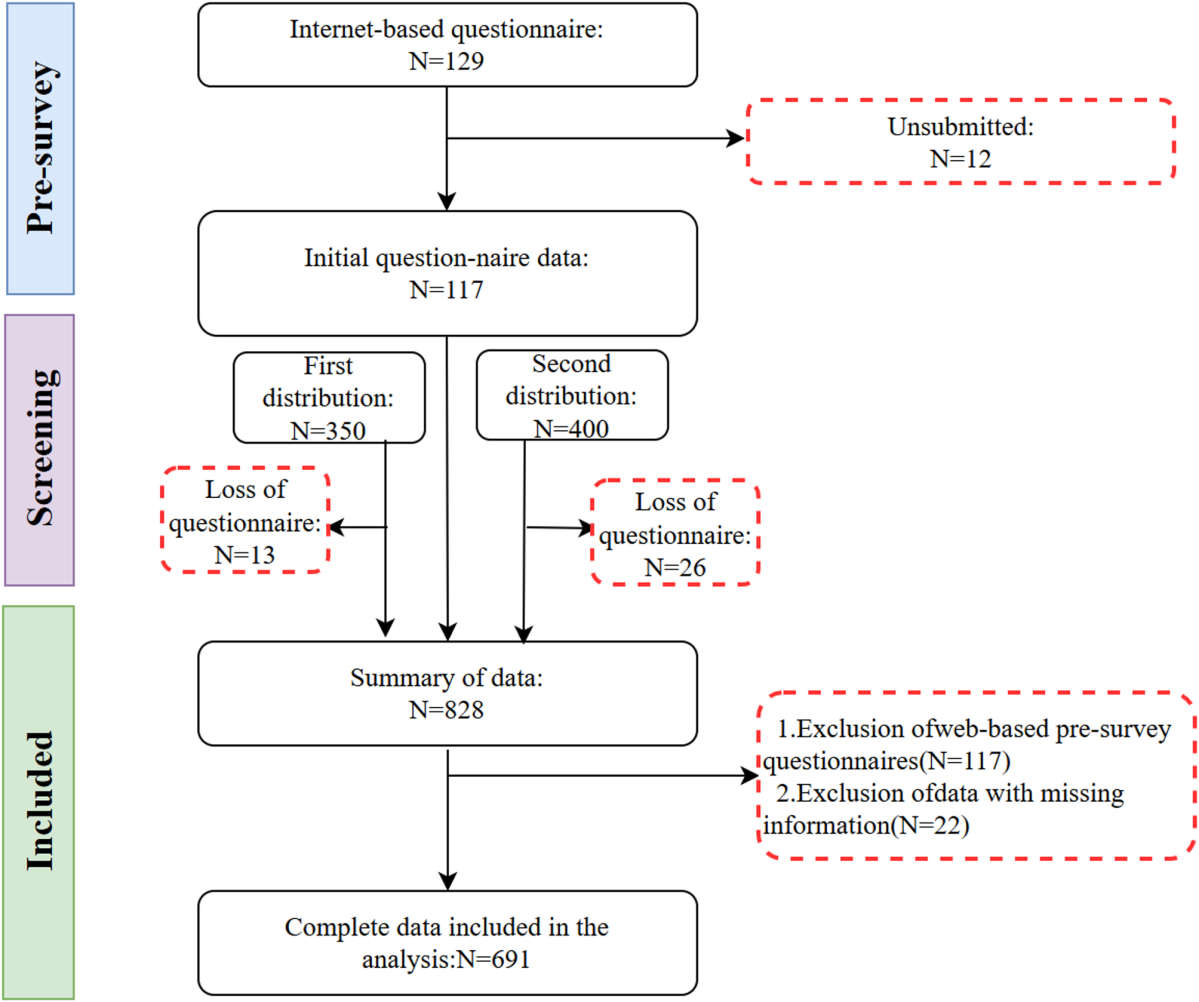
Participant flowchart.

Before the formal survey, this study conducted a pre-test with 120 medical students via an online questionnaire platform. Based on the results, survey items were revised to enhance the questionnaire’s reliability and validity. The data collection tool was generated using the “Question Star” platform. Informed consent was obtained from all participants before the survey commenced, and the questionnaire’s introduction page clearly outlined the research objectives and informed consent terms. This study employed a hybrid online-offline survey approach, yielding 710 completed questionnaires. After excluding invalid responses, 691 valid questionnaires were retained, resulting in a valid response rate of 97.3%. To assess the randomness of the missing data, Little’s MCAR test was conducted, and the results showed *p* > 0.05, indicating that the data were missing completely at random (MCAR). Therefore, for a small amount of missing data (< 5%), mean imputation was applied. This meets the minimum sample size requirement of the “10 times rule” for structural equation modeling (SEM)^54^. The research protocol was approved by the Biomedical Ethics Committee of the medical university. Additionally, Amos software was employed to mitigate the effects of data distortion and quantify relationships among constructs. Confirmatory factor analysis was conducted to assess the reliability and validity of the measurement model. Following confirmation of the research model and data fit, hypothesis testing was performed based on the analysis results of standardized factor loadings, path coefficients, and *p*-values. The participant flow is illustrated in Fig. 2.

Inclusion criteria for participants are as follows: (1) Currently enrolled as a full-time medical student in a medical program; (2) Conscious and alert, with no hearing, vision, or language communication impairments, and able to complete the questionnaire cooperatively; (3) Informed of the study’s purpose and voluntarily participating in the research. Exclusion criteria: (1) Individuals who refuse to participate in the study; (2) Individuals whose education is interrupted or who are unable to complete their studies during the survey period; (3) Non-medical students.

### Key variable measurement

All questionnaire items in this study were derived from existing literature and appropriately modified based on the established model and research objectives. The questionnaire comprises two sections. The first section collects respondents’ basic information, including grade level, gender, major, prior experience with AI products, duration of AI product usage, total internet usage time, internet learning time, and willingness to continue using AI products. Part Two comprises 11 variables and 47 measurement questions for the research hypotheses, with each variable having at least three questions. However, a preliminary survey indicated that simply replicating existing questionnaires would not fully accommodate the context and requirements of this study. Consequently, the research team conducted multiple revisions to enhance the questionnaire’s applicability, optimize item wording and structural design, and ensure the questionnaire accurately reflected the research theme while maintaining high validity and reliability. During the pre-survey phase, we analyzed items with low CIT values (Corrected Item-Total Correlation). When an item’s CIT value fell below 0.5 and its removal resulted in an increase in Cronbach’s alpha coefficient relative to the overall scale, we excluded that item to enhance the scale’s internal consistency. To ensure the validity of each dimension’s measurement, we redesigned some item statements, ensuring that each dimension retained at least three measurement items. All questionnaire items measuring each construct are displayed in Appendix. Each question was scored using a five-point Likert scale, with response options categorized as “Strongly Disagree,” “Disagree,” “Neutral,” “Agree,” and “Strongly Agree,” assigned values of 1, 2, 3, 4, and 5 respectively. Reverse scoring was applied to certain reverse-scored dimensions. Measurements for each dimension are calculated based on the average score of items within that dimension. After multiple rounds of refinement and validation, the final questionnaire demonstrated strong internal consistency, structural reliability, and convergent validity.

## Research results

### Data analysis and results

Statistical descriptions of the data were performed using SPSS 27.0 and Process (3.3.1) software, while Amos 27.0 software was employed to construct the research model. Student self-directed Learning Ability was treated as the outcome variable, with all other variables considered covariates. Based on this framework, the study introduced perceived interactivity to examine its influence on medical students’ intention to use DeepSeek. Performance expectancy and effort expectancy were incorporated as moderators to assess their impact on the relationship between perceived interactivity and behavioral intention. Origin (2024) software was employed for moderation effect analysis. Model fit was assessed using four fit indices: relative chi-square (x^2^/df), Pareto-optimized goodness-of-fit index (PGFI), goodness-of-fit index (GFI), and root mean square error of approximation (RMSEA). A lower relative chi-square value indicates less dependence on sample size and better model fit. Values between 1 and 2 are considered acceptable. PGFI and GFI range from 0 to 1, with values above 0.75 indicating a good fit. RMSEA measures the approximation between the confirmatory structure and modeling data; values below 0.08 indicate good model fit. A *p*-value < 0.05 is considered statistically significant.

### Demographic characteristics

**Fig. 3:**
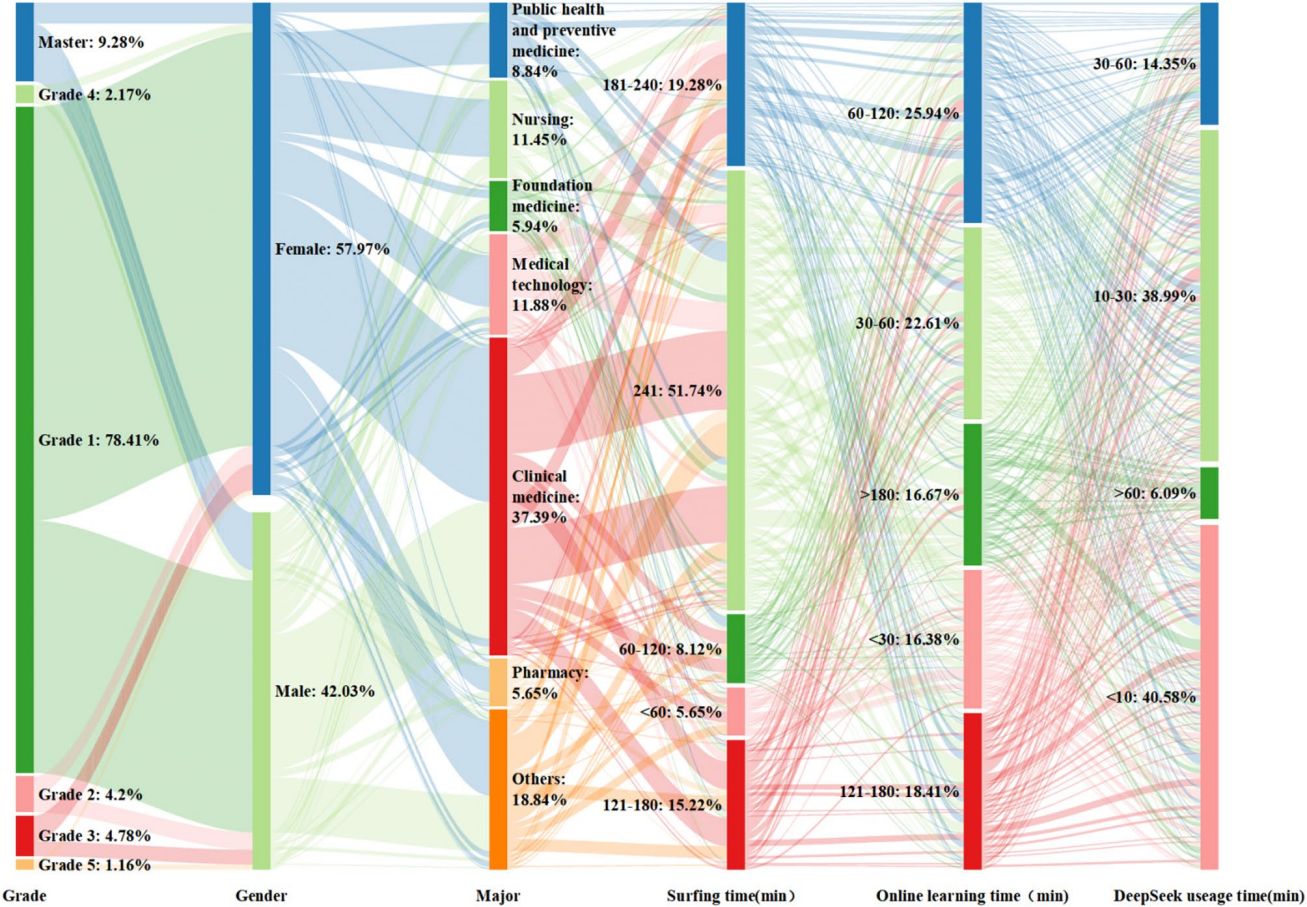
Demographic characteristics heat map.

This study analyzed data using Amos 27.0. Based on 691 valid questionnaire samples, the majority of participants were female (n = 406, 57.18%). First-year students constituted the largest cohort, the highest proportion of participants reported daily internet usage of ≥ 4 h (n = 372, 52.39%), while the largest group spent 1–2 h daily on online learning (n = 181, 25.49%). The majority indicated they would continue using AI products (n = 634, 89.3%). Overall, the survey sample structure was reasonable, facilitating model validation for AI product usage intent. Participant characteristics are shown in Fig. 3.

### Validity and reliability analysis

External model testing assesses reliability, overall validity, and discriminant validity. It requires standardized factor loadings exceeding 5 for each dimension. If an item’s factor loading fails to meet the recommended threshold, it indicates the item lacks representativeness and must be removed. All factor loadings in this study exceeded 0.5; all items were retained. Internal consistency across all dimensions was assessed using Cronbach’s Alpha and latent variable reliability. Typically, Cronbach’s Alpha should exceed 0.7. Higher CR (Construct Reliability) values for latent variables indicate greater item relevance within the construct. However, Hair et al. and Fornell and Larcker recommend CR values of 0.6 or higher. The Cronbach’s Alpha and CR values for all aspects of this study exceeded the recommended thresholds, indicating strong internal consistency for the model. Test results are presented in Table 1. Convergent validity measures the degree of convergence or correlation among multiple indicators within the same dimension. According to relevant literature, factor loadings for each dimension must exceed 0.7, construct reliability must exceed 0.6, and average variance extracted must exceed 0.5.

**Table 1:** Composite reliability and validity analysis.

Facet	Item	Parameter significance estimation				Factor loading	Composition validity	Convergence validity	Cronbach’s alpha
		Unstd	S.E	C.R	*p*	Std	CR	AVE	
TAC	v1	1.000				0.847	0.70	0.900	0.901
	v2	1.004	0.036	27.731	***	0.871			
	v3	0.897	0.035	25.819	***	0.827			
	v4	0.857	0.035	24.255	***	0.792			
TTF	V5	1.000				0.868	0.71	0.920	0.932
	V6	0.948	0.036	27.731	***	0.829			
	V7	0.989	0.033	30.006	***	0.847			
	V8	0.968	0.033	29.460	***	0.839			
	V9	0.948	0.033	28.996	***	0.831			
SI	V10	1.000			***	0.812	0.72	0.870	0.883
	v11	0.975	0.045	21.529	***	0.725			
	v12	1.024	0.040	25.868	***	0.826			
	v13	1.015	0.043	23.575	***	0.775			
PE	V14	1.000			***	0.853	0.78	0.900	0.711
	V15	0.972	0.039	24.989	***	0.793			
	V16	0.978	0.035	27.761	***	0.848			
	V17	0.952	0.037	25.646	***	0.807			
EE	V18	1.000			***	0.759	0.68	0.850	0.895
	V19	0.988	0.049	20.202	***	0.769			
	V20	1.047	0.054	19.538	***	0.746			
	V21	0.997	0.049	20.171	***	0.768			
PI	V22	1.000			***	0.844	0.73	0.920	0.916
	V23	0.978	0.038	25.881	***	0.815			
	V24	1.007	0.034	29.367	***	0.883			
	V25	0.992	0.034	29.364	***	0.883			
BI	V26	1.000			***	0.814	0.68	0.870	0.885
	V27	1.002	0.044	22.717	***	0.760			
	V28	1.038	0.045	22.983	***	0.766			
	V29	0.907	0.045	20.295	***	0.698			
	V30				***	0.754			
SE	V31	1.000			***	0.866	0.73	0.920	0.917
	V32	0.972	0.033	29.253	***	0.838			
	V33	0.956	0.032	30.266	***	0.853			
	V34	0.979	0.031	31.479	***	0.871			
SDLA	V35	1.000			***	0.814	0.71	0.900	0.911
	V36	0.936	0.041	22.889	***	0.763			
	V37	0.995	0.043	23.030	***	0.767			
	V38	0.930	0.041	22.810	***	0.762			
	V39	0.919	0.041	22.307	***	0.749			
	V40	1.053	0.042	25.157	***	0.816			
TEC	V35	1.000			***	0.844	0.79	0.930	0.932
	V36	0.971	0.036	26.849	***	0.813			
	V37	1.000	0.034	29.637	***	0.861			
	V38	1.027	0.038	27.262	***	0.820			
	V39	0.995	0.035	28.075	***	0.835			
	V40	1.088	0.041	26.790	***	0.812			

### Correlation analysis

Discrimination refers to the degree of differentiation between various aspects of a model. The greater the differentiation between aspects, the lower their correlation, indicating higher discrimination between them. According to validity assessment methods, when the square root of AVE on the diagonal is substantially greater than the correlation coefficients with other factors, it indicates that validity standards are met. As shown in the figure, the model in this study exhibits relatively high validity, with results presented in Table 2.

**Table 2:** Variable correlation analysis.

Variables	AVE	PE	PI	SI	TTF	TEC	TAC	SE	BI	SDLA	EE
PE	0.900	0.948									
PI	0.920	.743**	0.959								
SI	0.870	.769**	.805**	0.933							
TTF	0.920	.794**	.845**	.829**	0.959						
TEC	0.930	.769**	.826**	.808**	.894**	0.964					
TAC	0.900	.652**	.737**	.736**	.762**	.766**	0.948				
SE	0.920	.782**	.800**	.822**	.853**	.845**	.761**	0.959			
BI	0.870	.718**	.762**	.778**	.788**	.808**	.793**	.851**	0.933		
SDLA	0.900	.727**	.770**	.784**	.806**	.810**	.762**	.860**	.867**	0.948	
EE	0.850	.778**	.803**	.768**	.799**	.778**	.688**	.766**	.743**	.741**	0.922

**At the 0.01 level (2 tailed), the correlation was significant.

### Structural equation modeling testing

Using AMOS software to analyze the structural equation model in Fig. 1 yielded standardized path coefficients (Table 3). Structural equations feature various fit indices that measure the degree of alignment between questionnaire data and the structural equation model. Typically, an acceptable fit is indicated by CMIN/df < 5, CFI < 5, CFI, GFI, IFI, and NFI ≥ 0.7, and RMSEA < 0.1. As observed, all model fit indices are acceptable or good (CMIN/df = 3.647,PGFI = 0.921,GFI = 0.896,CFI = 0.919,RMSEA = 0.065). Therefore, the constructed structural equation model demonstrates a high degree of fit with the actual questionnaire data, and the obtained data better explain the relationships among variables within the model.

**Table 3:** Path coefficient table.

Path			Non-standardized path	Standardized path	S.E	C.R	*p*	Conclusion
PE	< –-	TEC	0.764	0.818	0.034	22.410	***	Support
PE	< –-	PI	0.158	0.180	0.024	6.694	***	Support
TTF	< –-	TAC	0.068	0.082	0.014	4.841	***	Support
TTF	< –-	TEC	0.979	0.971	0.034	28.782	***	Support
SI	< –-	TEC	0.958	0.957	0.038	25.407	***	Support
EE	< –-	PI	0.322	0.428	0.025	12.825	***	Support
EE	< –-	TEC	0.293	0.367	0.045	6.467	***	Support
EE	< –-	PE	0.333	0.390	0.051	6.560	***	Support
BI	< –-	TTF	− 0.004	− 0.004	0.076	− 0.047	0.042	Support
BI	< –-	EE	0.122	0.104	0.063	1.936	**	Support
SE	< –-	SI	1.034	0.974	0.039	26.768	***	Support
BI	< –-	PE	0.072	− 0.071	0.048	1.500	0.034	Support
BI	< –-	PI	0.041	0.046	0.028	1.481	0.039	Support
BI	< –-	SI	0.871	0.925	0.083	10.511	***	Support
SDLA	< –-	SE	0.518	0.575	0.161	3.213	0.001	Support
SDLA	< –-	SI	− 0.434	− 0.453	0.203	− 2.136	0.033	Support
SDLA	< –-	BI	0.879	0.865	0.102	8.590	***	Support

***p < 0.001.

### Regression analysis

**Fig. 4:**
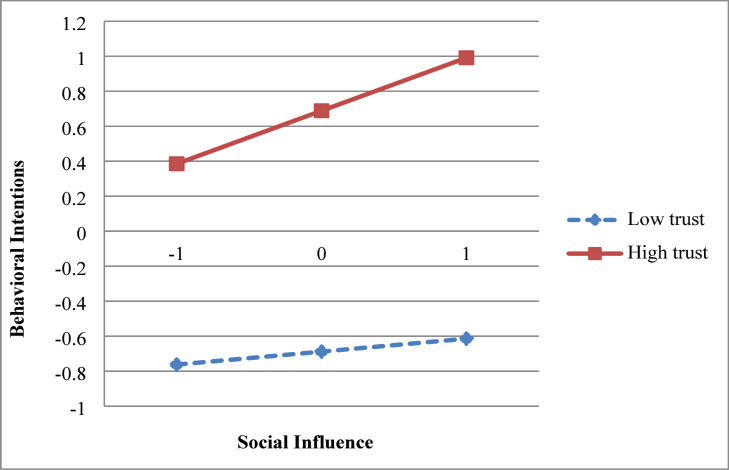
Modulation effect diagram.

Based on H10 and H11, this study divided the high-trust and low-trust groups by an average of one standard deviation above and below the mean, respectively, and plotted an intuitive slope diagram as shown in Fig. 4. The resulting standard errors and 95% confidence intervals are presented in Table 4. The findings indicate that medical students’ trust in DeepSeek significantly and positively influences their intention to use DeepSeek (β = 0.0075, *p* < 0.05). Similarly, social influence significantly and positively impacts students’ intention to use DeepSeek (β = 0.0039, *p* < 0.05). Therefore, this hypothesis is supported.

**Table 4:** Moderating effects.

Variables	S.E	95%CI	
SI → T → BI	0.0152	3.9383	3.9981

### Mediation analysis

**Fig. 5:**
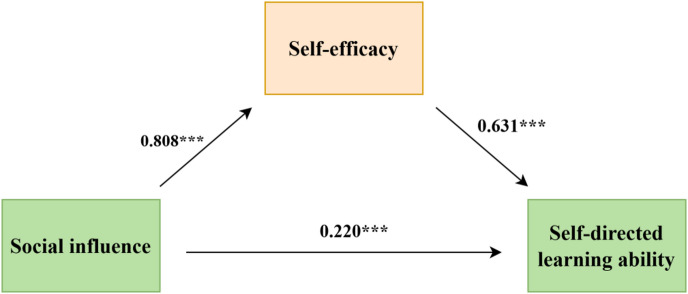
Mediation model diagram.

To explore the underlying mechanism of the significant positive influence of social influence on self-directed learning ability, self-efficacy was further introduced as a mediating variable in the structural equation model. The mediation effect was tested using Model 4 in the SPSS macro Process, and the mediating role of self-efficacy between social influence and self-directed learning ability was verified according to the method provided by Hayes (Fig. 5, Table 5).

**Table 5:** Mediation model path analysis.

Mediation path	Effect size	SE	Bootstrap 95% CI	Proportion of mediating effect
Total effect	0.730	0.022	0.687,0.773	
Direct effect	0.220	0.031	0.160,0.280	30.137%
Indirect effect	0.510	0.034	0.442,0.577	69.863%

## Discussion

### The core driving role of Deepseek in perceived interactivity

**Fig. 6:**
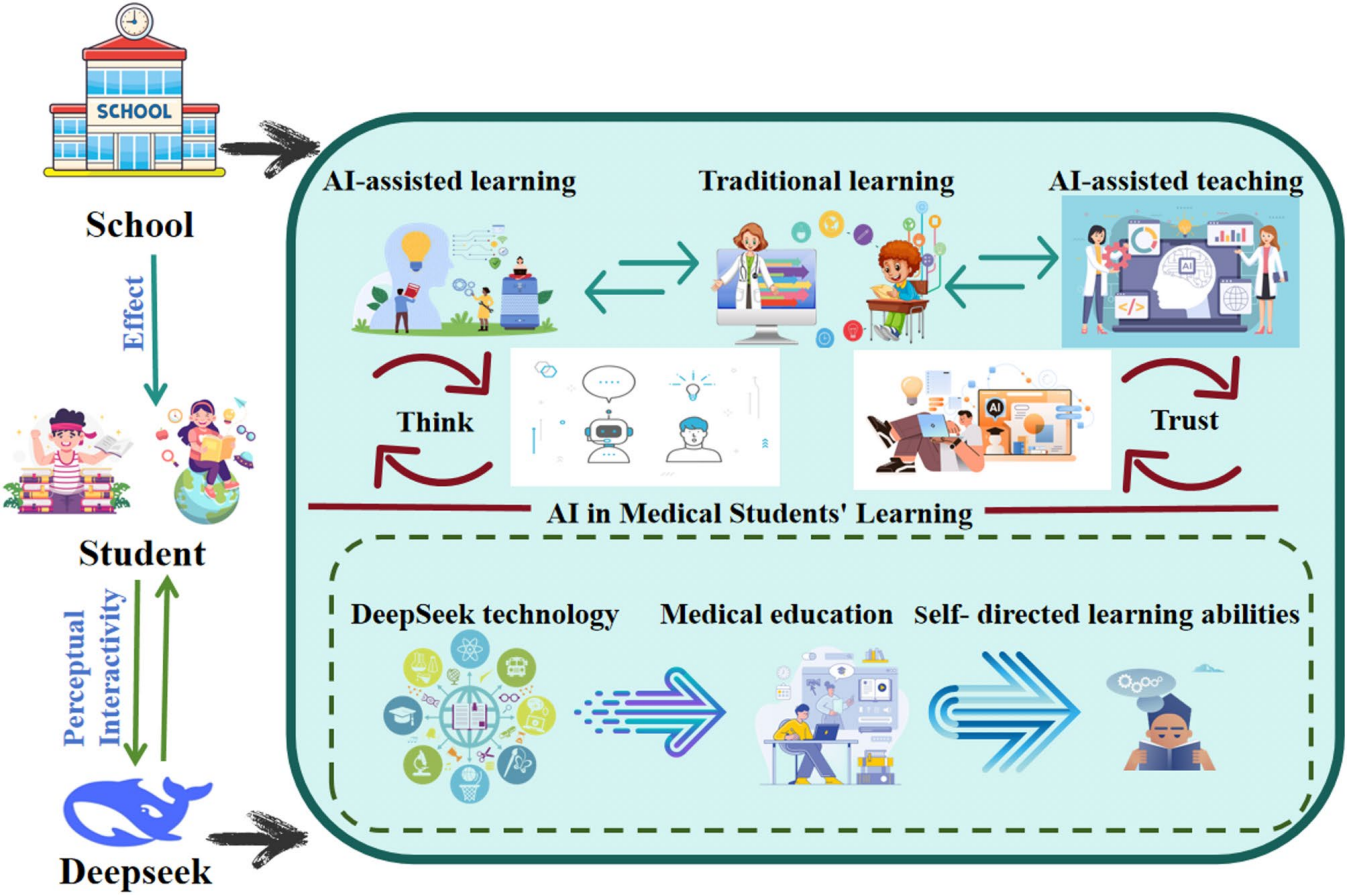
Correlation between DeepSeek and autonomous learning capabilities.

Perceived interactivity refers to users’ subjective perception of DeepSeek’s real-time feedback, personalized responses, and bidirectional communication capabilities^55^. It significantly influences medical students’ willingness to adopt the technology through performance expectations and effort expectations (see Fig. 6). Findings indicate that DeepSeek, through instant Q&A and semantic understanding, not only enhances students’ learning efficiency but also strengthens their sense of control and feedback timeliness, thereby further stimulating learning immersion and engagement. In complex medical diagnostic tasks, DeepSeek’s perceived interactivity exhibits stronger motivational effects^56^, with students tending to view it as a “virtual mentor.” This characteristic aligns closely with the collaborative learning emphasis inherent in medical education^57^. This finding extends the UTAUT model, revealing the unique value of human-like interaction in educational settings and offering a new theoretical perspective for technology adoption research.

### The dominant driver of social impact

This study found that social influence exhibited the strongest direct effect, thereby validating the central role of social norms in the UTAUT model^58^. In medical education settings, social factors such as faculty recommendations and peer modeling proved more effective in motivating students to adopt DeepSeek than the tool’s functional attributes (e.g., efficiency gains)^59^. As reported by scholar Nisar Ahmed Dahri, this indicates that social influence played a role in their decision to explore AI-driven learning tools. Our survey supports this hypothesis, highlighting the impact of social influence on educators’ attitudes toward adopting AI technologies. Many surveyed educators stated they use AI technologies due to positive peer experiences^60^. This outcome likely stems from the medical field’s cultural emphasis on social recognition and collaboration, where students demonstrate heightened sensitivity to authority and peer endorsement. However, the negative effect of social influence indicates that overreliance on external evaluations may undermine students’ learning initiative and self-regulation abilities. This dual effect suggests that while leveraging social influence to promote technology adoption, attention should be paid to its potential inhibitory effects on autonomous learning capabilities^61^.

### The psychological empowerment mechanism of self-efficacy

Perceived Interactivity indirectly promotes self-directed learning by enhancing self-efficacy, thereby supporting the social cognitive theory hypothesis regarding technology-enabled psychological construction^62^. DeepSeek’s interactive features—such as instant feedback and guided prompts—help medical students build confidence while navigating complex medical knowledge. For instance, AI-assisted case exercises enable students to progressively master diagnostic reasoning, thereby strengthening their inclination toward autonomous exploration in unfamiliar domains. However, negative social influence effects indicate that overreliance on AI interactions may foster cognitive inertia and diminish intrinsic learning motivation^63^. This finding aligns with research on technology dependency risks^64^, underscoring the necessity of balancing technology-enabled learning with the cultivation of autonomous learning capabilities.

### The moderating effect of trust

AI trust is defined as users’ positive belief in the reliability, safety, and intent of artificial intelligence systems. Particularly in high-risk or specialized application scenarios (such as medical education), trust becomes a critical psychological boundary condition for technology adoption and sustained use^65,66^. Trust enhances users’ identification with and reliance on AI, thereby elevating expectations and motivation during the learning process. Higher performance expectations further incentivize users to maintain interest and intent in using AI. If users believe AI can significantly improve academic achievement, they are more likely to continue using it^67^. Research indicates that both emotional attachment and trust reduce users’ skepticism toward technology, strengthen their dependence on it, and consequently boost learning motivation and willingness for sustained use^68^. This study finds that AI trust significantly amplifies the positive effect of social influence on usage intention. Specifically, in groups with higher trust levels, peer or teacher recommendations exert a more pronounced driving force on DeepSeek adoption. This result aligns with recent research on trust mechanisms in AI education, indicating that trust not only alleviates users’ concerns about technological uncertainty but also strengthens the shaping power of social norms on behavioral intentions^69^. Furthermore, when college students exhibit low interpersonal trust levels, the impact of teachers’ emotional support on their academic self-efficacy diminishes; conversely, this influence is amplified when interpersonal trust is high^70–72^.

### Comparison of Deepseek with other AI tools

To place the findings of this study within a broader academic context, we conducted a comparative analysis with generative AI tools supported by existing empirical research in the literature, such as ChatGPT. Existing research indicates that ChatGPT can effectively promote personalized learning in medical education, enhance student engagement and self-efficacy^73^, and improve academic performance through automated grading and instant feedback mechanisms. However, its reliance on cloud computing has raised concerns about data privacy^74^, which could adversely affect users’ long-term trust and knowledge retention outcomes.

Comparative analysis demonstrates that DeepSeek exhibits significant advantages across multiple dimensions. It provides more comprehensive information and more timely updates when generating patient education guides^75^. Its on-premises deployment capability effectively safeguards data privacy and security^76^, eliminating potential risks associated with cloud-based data transmission. This feature makes it particularly suitable for data-sensitive environments such as hospitals. Moreover, its open-source nature significantly enhances the platform’s adaptability and scalability for diverse medical tasks^77^. These findings not only provide strong support for the results regarding interactivity and self-efficacy in this study, but also highlight DeepSeek’s dual value in advancing the digital transformation of medical education—balancing technological empowerment with the preservation of data sovereignty.

## Conclusion

At a critical juncture in the digital transformation of medical education, DeepSeek has pioneered an innovative pathway to enhance medical students’autonomous learning capabilities by reconfiguring human–machine interaction models. This initiative not only deepens our understanding of the efficacy mechanisms of generative AI in education but also underscores the importance of synergistic collaboration among “technology acceptance, cognitive empowerment, and contextual adaptation” in medical education’s digital transformation. Consequently, it establishes a robust theoretical foundation for subsequent research and practice.

### Theoretical contributions

This study integrates the UTAUT, SCT, and TTF frameworks to construct a multidimensional theoretical model that systematically reveals the mechanism through which the perceived interactivity of generative AI (DeepSeek) influences medical students’autonomous learning abilities. This framework not only expands the application boundaries of the UTAUT model in educational technology but also deepens our understanding of the complex relationship between technology adoption and learning outcomes by integrating social cognitive perspectives with the task-technology fit perspective. Findings indicate that social influence, as the core driver of technology adoption, exerts a significantly stronger effect than the indirect roles of performance expectations and effort expectations. Furthermore, self-efficacy plays a significant mediating role between technology behavioral intention and self-directed learning ability, further validating social cognitive theory’s explanatory power regarding self-efficacy in predicting behavioral outcomes.

### Practical contributions

The empirical findings of this study provide multi-level, actionable practical insights for the application of DeepSeek in medical education. For educational and healthcare institutions, efforts should focus on building socially interactive AI platforms. For instance, a virtual mentor system based on DeepSeek could be developed to simulate real-time clinical scenarios through dialogue, enabling students to engage in role-playing during group learning and receive immediate feedback to enhance collaborative learning outcomes. Concurrently, differentiated curriculum integration solutions should be designed, such as embedding DeepSeek into problem-based learning (PBL) modules. In this scenario, instructors can upload anonymized real-world case data to guide students in using DeepSeek to generate diagnostic hypotheses and treatment plans. Personalized feedback facilitates their transition from passive knowledge recipients to active explorers. In exam preparation courses, DeepSeek functions as an adaptive question bank tool. It dynamically adjusts question difficulty and content based on students’ response history, provides targeted explanations and literature support, and simulates the clinical reasoning component of the National Medical Licensing Examination. This approach enhances students’ self-efficacy and exam pass rates.

Additionally, a dynamic evaluation system based on interactive data can be established. By leveraging DeepSeek’s log analysis capabilities to track student learning trajectories, it generates visual reports that provide data support for teachers to optimize teaching strategies. These concrete and actionable recommendations not only address the tension between the dominant effects of social influence and the suitability of medical specialties, but also offer a systematic, evidence-based solution pathway for the deep integration of intelligent technology with medical education. This holds significant reference value for advancing digital transformation in this field.

### Limitation and suggestions for future research

Despite the significant findings of this study, several limitations remain. First, the sample primarily consists of Chinese medical students, and geographical and cultural differences may limit the external validity of the results. Second, as a specific generative AI tool, DeepSeek’s interactive features may not fully reflect the common characteristics of other AI systems. Inherent differences among AI systems in interaction design, privacy protection mechanisms, and technical architecture may significantly influence users’ perception of interactivity and their subsequent usage behavior. Furthermore, data collection for this study was primarily conducted using self-report questionnaires. Although all scales underwent standard reliability and validity testing, the potential influence of social desirability bias and common method bias cannot be entirely ruled out. These factors may impose certain limitations on the accuracy of the measurement results.

Future research may adopt longitudinal designs to track the long-term evolution of medical students’ AI usage behaviors, or expand to other professional fields for cross-cultural comparisons. It should also explore the impact of emerging AI technologies—such as multimodal interaction—on autonomous learning to deepen the integration of theory and practice in educational technology.

## Data Availability

The datasets used and/or analysed during the current study available from the corresponding author on reasonable request.

## Appendix

**Table:**

Observed variables	Indicator code	Observed indicator content	References
Performance expectancy (PE)	PE1	I find DeepSeek helpful for my studies	Udo et al.^78^
	PE2	Using DeepSeek for learning meets my daily needs	
	PE3	DeepSeek helps me complete learning tasks faster	
	PE4	Using DeepSeek makes my life more convenient	
Effort expectancy (EE)	EE1	Learning how to use DeepSeek was easy for me	Udo et al. ^78^
	EE2	I can use DeepSeek proficiently	
	EE3	DeepSeek’s operation steps are clear and easy to understand	
	EE4	I find DeepSeek very easy to use	
Perceived interactivity (PI)	PI1	DeepSeek accurately understands my questions	Wang et al.^79^
	PI2	DeepSeek provides comprehensive answers to my queries	
	PI3	DeepSeek offers multiple interaction methods	
	PI4	DeepSeek’s interaction design is quite flexible	
Social influence (SI)	SI1	My teacher believes I should use DeepSeek	Sun et al.^80^
	SI2	My friends and classmates think I should use it	
	SI3	My family thinks I should use DeepSeek	
	SI4	My peers are all using DeepSeek for learning	
Task-Technology fit (TTF)	TTF1	I believe DeepSeek’s capabilities are sufficient to support my learning activities	Wang et al.^79^
	TTF2	DeepSeek provides adequate assistance for coursework	
	TTF3	DeepSeek’s information retrieval functions are sufficient	
	TTF4	DeepSeek’s text generation capabilities are sufficient	
	TTF5	DeepSeek offers innovative perspectives	
Technology characteristics (TEC)	TEC1	DeepSeek delivers ubiquitous services in education	Zhou et al.^81^
	TEC2	DeepSeek provides reliable and efficient services	
	TEC3	DeepSeek offers real-time services	
	TEC4	DeepSeek can provide features relevant to course learning	
	TEC5	DeepSeek can deliver a personalized search experience	
Task characteristics (TAC)	TAC1	I need to use DeepSeek anytime, anywhere to access information	Zhou et al.^81^
	TAC2	I need to utilize DeepSeek for text generation anytime, anywhere	
	TAC3	I need to utilize DeepSeek for coursework ideas anytime, anywhere	
Trust (T)	T1	I believe using DeepSeek in education benefits me	Shahzad et al.^82^
	T2	I use DeepSeek during my studies	
	T3	I believe society should support DeepSeek education	
	T4	DeepSeek should enter the education sector	
	T5	I look forward to authentic information about DeepSeek in education	
Self-efficacy (SE)	SE1	I find DeepSeek reliable	Shahzad et al.^82^
	SE2	I trust that using DeepSeek aids learning	
	SE3	Using DeepSeek has saved me a lot of time	
	SE4	I am interested in learning with DeepSeek	
Behavioral intention (BI)	BI1	I am willing to work hard to acquire knowledge and skills related to DeepSeek	Wang et al.^12^
	BI2	Using DeepSeek is an important part of my learning life	
	BI3	I will always use DeepSeek in my studies	
	BI4	I plan to use DeepSeek frequently for learning	
	BI5	I intend to continue using DeepSeek for learning in the future	
Self-directed learning abilities (SDLA)	SDLA1	DeepSeek enhances my learning capabilities	Timothy et al.^83^
	SDLA2	DeepSeek improves my study methods	
	SDLA3	DeepSeek supports my independent learning	
	SDLA4	DeepSeek strengthens my self-directed learning skills	

## Abbreviations

SEM: Structural equation modeling
UTAUT: Unified theory of acceptance and use of technology
SCT: Social cognitive theory
TTF: Task-technology fit
CNMLE: China national medical licensing examination
T: Trust
PI: Perceived interactivity
GFI: Goodness-of-fit index
RMSEA: Root mean square error of approximation
PGFI: Pareto-optimized goodness-of-fit index
CR: Construct reliability
AI: Artificial intelligence
PE: Performance expectancy
EE: Effort expectancy
SI: Social influence
BI: Behavioral intention
TAC: Task characteristic
TEC: Technological characteristic
SE: Self-efficacy
SDLA: Self-directed learning ability
